# PET and SPECT Imaging of ALS: An Educational Review

**DOI:** 10.1155/2023/5864391

**Published:** 2023-08-19

**Authors:** Ayaan M. Jamali, Manasa Kethamreddy, Brian J. Burkett, John D. Port, Mukesh K. Pandey

**Affiliations:** Department of Radiology, Mayo Clinic, Rochester, MN, USA

## Abstract

Amyotrophic lateral sclerosis (ALS) is a disease leading to progressive motor degeneration and ultimately death. It is a complex disease that can take a significantly long time to be diagnosed, as other similar pathological conditions must be ruled out for a definite diagnosis of ALS. Noninvasive imaging of ALS has shed light on disease pathology and altered biochemistry in the ALS brain. Other than magnetic resonance imaging (MRI), two types of functional imaging, positron emission tomography (PET) and single photon emission computed tomography (SPECT), have provided valuable data about what happens in the brain of ALS patients compared to healthy controls. PET imaging has revealed a specific pattern of brain metabolism through [^18^F]FDG, while other radiotracers have uncovered neuroinflammation, changes in neuronal density, and protein aggregation. SPECT imaging has shown a general decrease in regional cerebral blood flow (rCBF) in ALS patients. This educational review summarizes the current state of ALS imaging with various PET and SPECT radiopharmaceuticals to better understand the pathophysiology of ALS.

## 1. Introduction

Amyotrophic lateral sclerosis is a disease that damages upper and lower motor neurons in the brain and spinal cord, leading to progressive neurodegeneration [1]. ALS generally starts in the limbs, known as limb-onset ALS, although there is bulbar-onset ALS, which has facial symptoms, such as difficulty in eating and speaking. These symptoms can progress to more serious complications, such as affecting respiration, leading to death [1, 2]. The incidence of ALS varies by age and region but is roughly anywhere from 1 to 3 cases per 100,000 people, while the prevalence sits at about 3-6 cases per 100,000 people [1, 3–5]. ALS has a poor prognosis, with an average survival time of 3-5 years after symptoms appear [1], although there is a particular ALS phenotype that confers longer survival for more than a decade [6]. ALS can be split into two types: familial (fALS) and sporadic (sALS). In familial cases, there is a history of ALS in the patient's family. These cases make up about a tenth of ALS while sporadic cases take up the rest [1].

ALS is a difficult disease to diagnose. There is a delay from onset of ALS to clinical diagnosis, which can be more than a year [1, 4]. There are criteria used for ALS diagnosis such as the El Escorial criteria and the Awaji criteria, which both rely on the presence of upper motor neuron signs and lower motor neuron signs (of degeneration). Additional scales and rankings can assess the extent of such lower or upper motor impairment. With these assessments, many studies have correlated imaging patterns with the severity of the lower or upper neuron signs, and this will be discussed at times in the review. However, some patients exhibit only upper motor neuron signs or lower motor neuron signs. These are subtypes of ALS and classified under a different name. In the case of only upper motor neuron signs, patients are diagnosed with primary lateral sclerosis (PLS), while those with only lower motor signs are diagnosed with progressive muscle atrophy (PMA) [4].

When ALS is diagnosed, it can be categorized into various phenotypes such as bulbar or limb onset, ALS with frontotemporal dementia (ALS-FTD—making up to 15% of cases) [7], and ALS stemming from a particular faulty gene or protein. Examples of these proteins (and their respective genes) include TAR DNA binding protein 43 (TDP-43), fused in sarcoma (FUS), superoxide dismutase-1 (SOD1), and other additional proteins [8, 9]. These faulty proteins can aggregate to form inclusions that harm neurons (as seen in many other neurodegenerative diseases as well). It is suggested that these inclusions can disrupt cellular activity by interacting with other proteins or interrupting certain molecular pathways [1, 8]. TDP-43 aggregates, especially, have been found in a substantial majority of ALS cases, in more than 95% [7]. Furthermore, another gene known as C9ORF72 (chromosome nine, open reading frame 72) has been implicated as being the most common genetic mutant in ALS with GGGGCC hexanucleotide repeat expansions in a noncoding region of the gene [10–12]. Different mutations occur in varying frequencies by gene, but nevertheless, data has still been compiled by gene. TARDBP (TDP-43) and FUS mutations make up less than 5% of ALS cases, while SOD1 mutations comprise up to 20% of fALS cases, and C9ORF72 mutations can be up to half of fALS [7, 8]. Different imaging studies have classified ALS subjects into the several phenotypic categories described above, and this review will discuss patterns (particularly metabolic ones) found in many of these mutants.

Aside from protein aggregation, another mechanism of neurodegeneration is neuroinflammation caused by astrocytes and microglia. Activated glia can release cytokines, thus allowing T cells to penetrate the brain, harming neurons, and leading to degeneration [13, 14]. Several biomarkers of neuroinflammation have been identified, such as the 18 kDa (kilodalton) translocator protein (TSPO), monoamine oxidase B enzyme (MAO-B), and cyclooxygenase (COX) enzymes [15]. As a result, numerous studies, many of which will be discussed in this review, have targeted and imaged these biomarkers in ALS patients.

In addition to the neuroinflammation and protein aggregation mentioned above, metabolic dysregulation in ALS occurs, with evidence in the literature of pathologic changes in cellular metabolism as well as systemic changes in metabolism in the central nervous system and throughout the body. Patients with ALS may experience global hypermetabolism and weight loss, with body weight being an important prognostic factor in the diagnosis [16]. Cellular metabolic changes in ALS have been described in the GLUT1 and neuronal GLUT3 glucose transporters, glycolysis, the citric acid cycle, the pentose phosphate pathway, and oxidative phosphorylation. Consequences of these abnormalities in the central nervous system such as mitochondrial dysfunction, increased oxidative stress, glutamate excitotoxicity, and derangements in neuroglial interactions may yield neuronal dysfunction and degeneration [16]. Much of the literature investigating in vivo central nervous system metabolism in ALS uses [^18^F]FDG PET to visualize and quantify glucose transport and metabolism. As a result, a large portion of this review is dedicated to summarizing alterations to glucose metabolism in ALS, with several studies contrasting patients with controls to identify brain regions with changed metabolism.

As ALS pathology is still being researched, there is no specific biomarker or hallmark of ALS, and thus, multiple tests such as electromyography, imaging, and genetic testing are utilized for a comprehensive diagnosis [4]. With respect to imaging in particular, various modalities have been used to diagnose and better understand ALS. These include magnetic resonance imaging (MRI), single photon emission computed tomography (SPECT), and positron emission tomography (PET). PET and SPECT are of particular interest due to their functional imaging nature using novel radiotracers to target specific biological pathways or biochemistry involved in ALS pathology. Such imaging can aid in diagnosing and staging ALS, especially in light of the long time to diagnosis. The purpose of this review is to summarize the current state of ALS imaging with various PET and SPECT radiopharmaceuticals, to outline gaps in the current knowledge of ALS, and to plan a better approach to understand the pathophysiology of ALS.

### 1.1. Basics of PET Imaging and Its Relevance in ALS

**Figure 1:**
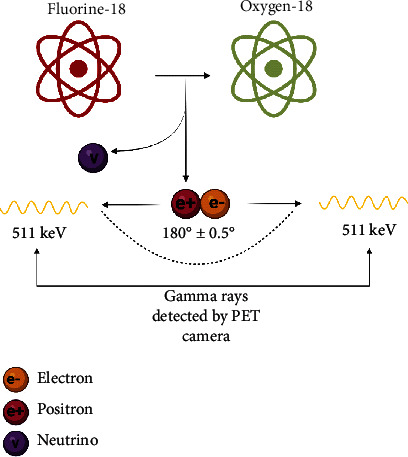
^18^F decays into ^18^O, releasing a positron (along with a neutrino) in the process which can collide with an electron to produce detectable gamma rays.

PET imaging relies upon radionuclides, which decay by converting a proton to a neutron, releasing a positron in the process. The newly produced positron can collide with a nearby electron, canceling them both (called annihilation) and releasing high energy gamma rays (two photons of 511 keV at an approximately 180° angle) in the process. These photons can be detected by the PET scanner to produce an image [17]. PET imaging is highly sensitive due to the high energy gamma rays. Because PET is an imaging modality that provides in vivo functional information, it is a sensitive tool for detecting pathologic changes in function that may occur prior to disease-related anatomic and structural abnormalities. For example, PET is capable of visualizing and quantifying physiologic processes including perfusion, metabolism, or protein synthesis, and PET can be used to visualize the distribution of receptors and molecular binding sites such as those for neurotransmitters. In clinical use, PET is typically spatially coregistered with structural imaging obtained with CT or MRI, giving detailed information about the location of functional abnormalities. Functional imaging and high sensitivity of PET makes it an extremely valuable tool for early detection of various diseases. An example of a commonly used radioisotope, [^18^F], is provided in Figure 1. PET radionuclides can be appropriately conjugated or covalently linked with molecules designed for specific biological targets. Once these radiolabeled molecules bind to specific proteins or accumulate in a specific brain region, it can demonstrate abnormality with respect to healthy controls. Information obtained via PET imaging may support a diagnosis of ALS.

Given the sensitivity and selectivity of PET probes, various PET radiotracers have been employed to better understand the pathophysiology of ALS. Among these, [^18^F]FDG is the predominantly used radiotracer to measure glucose metabolism in various brain regions. Other than hypometabolism and hypermetabolism of glucose in various brain regions of ALS, neuroinflammation is another pathological condition that has been implicated in ALS. As a result, various radiotracers measuring the degree and regions of neuroinflammation in ALS have also been applied to shed further light on the pathophysiology of ALS. Additionally, loss of neuronal density has been associated with ALS; therefore, various radiotracers measuring the synaptic density have been employed to measure the loss of neuronal density in ALS patients to better understand the progression of ALS. Finally, while not as frequent, a few radiotracers have been employed to image protein aggregation in ALS.

## 2. Application of Various PET Probes in ALS

As molecular imaging modalities, PET and SPECT are fundamentally functional imaging, meaning they provide specific information about a particular biochemical or cellular process, unlike the anatomical information provided by other imaging modalities (e.g., CT or MRI). There are several PET probes which have been developed to garner specific biochemical information in ALS pathophysiology, and they can be broadly categorized in following groups based on the information they provide:

- Glucose metabolic imaging of ALS
- Neuroinflammation imaging of ALS
- Synaptic or neuronal density imaging of ALS
- Protein aggregate imaging of ALS

### 2.1. Glucose Metabolic Imaging of ALS

Proper functioning of any organ, tissue, or cells requires a constant source of energy, and glucose is the primary source of energy to the brain. Any abnormalities in the brain often manifest in alteration of the rate of glucose metabolism, which may be specific to a single or multiple brain regions. Imaging this altered glucose metabolism in specific brain regions allows for comparison with controls to highlight affected parts of the brain under a particular disease condition. The mainstream radiotracer for glucose metabolic studies is 2-deoxy-2-[^18^F]fluoro-D-glucose, also known as [^18^F]FDG. It is a glucose analog and well suited for assessing brain glucose metabolism since the brain is a center of high metabolism. [^18^F]FDG is not fully metabolized but rather ends up as an intermediate in glycolysis, where its levels can be quantified to measure hypermetabolism or hypometabolism [16].

Several studies have investigated glucose metabolism in ALS, which has resulted in a characteristic pattern of both hypermetabolism and hypometabolism in certain parts of the brain. Furthermore, the pattern of altered glucose metabolism was demonstrated to vary in different ALS phenotypes. In this section, we highlight a general trend of glucose metabolism in different parts of the brain under ALS condition.

In clinical imaging studies of ALS, there is overwhelming evidence of glucose hypometabolism in the frontal lobe [18–22] and, more specifically, the motor cortices [23, 24]. Within the frontal lobe, the prefrontal cortex [23, 25], frontal eye fields [23], superior frontal gyrus [26–28], middle frontal gyrus [26–28], inferior frontal gyrus [26, 27], medial frontal gyrus (only the left side in one study) [24, 28], gyrus rectus [26, 27], and left precentral gyrus [28, 29] have been identified as regions of significant hypometabolism. The involvement of extramotor frontal lobe regions is in keeping with the clinical presentation of ALS in which a spectrum of cognitive and behavioral impairment can occur, including decline in executive functions of organization and planning, language, and personality changes, reflecting frontal lobe dysfunction [30]. The more distributed regions of frontal hypometabolism would also be expected in ALS patients meeting criteria of comorbid frontotemporal lobar degeneration (FTLD), with a well-characterized clinical association between FTLD and ALS likely reflecting the underlying molecular pathophysiology of these conditions related to TDP-43 proteinopathy. Impairment in frontal lobe executive functions and language fluency have been reported in up to 35% of ALS without dementia [31]. Hypometabolic motor regions include the premotor cortex [19, 23, 25, 29], primary motor cortex [32], and supplementary motor area [26, 27]. Aside from frontal and motor areas, other areas having been identified as hypometabolic include cingulate cortex regions (anterior, medial, paracingulate, and right posterior) [26–28], the thalamus [24, 28, 33], the basal ganglia [33], and the caudate nucleus [18].

**Figure 2:**
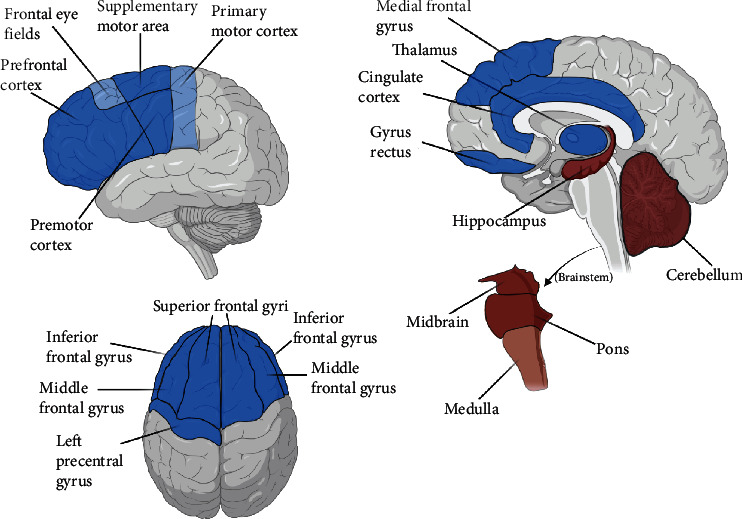
Brain regions in ALS with hypometabolism (shaded in blue) and hypermetabolism (shaded in red). The more transparent (lighter) blue and red regions indicate single study results. Note that the parahippocampal gyrus and basal ganglia, areas of reported hypometabolism in a few studies, are not depicted in the illustrated diagram.

With regard to glucose hypermetabolism in ALS, a few regions have been consistently identified by studies. The cerebellum [18, 19, 21, 24, 25, 27–29, 33] and hippocampus [18, 23–25] are frequently listed as regions with hypermetabolism. Additionally, the brainstem, including regions such as the midbrain [18, 23, 29, 34], pons [29, 34], and medulla [34], is also listed. One other region identified as hypermetabolic is the parahippocampal gyrus [27]. Hypermetabolism may seem unexpected, given that ALS consists of neuron degeneration. However, many studies have theorized that hypermetabolism could stem from a high presence of activated microglia and astrocytes [18, 19, 23, 29, 34]. Astrocytes are responsible for a significant portion of the brain's metabolism; thus, astrocytosis has been implicated as causing hypermetabolism. The major metabolic findings described in the above two paragraphs are summarized in Figure 2.

The degree of metabolic abnormalities in ALS identified on PET is heterogenous, which we speculate to be reflective of varying degrees of disease severity. For example, prognosis and cognitive performance in ALS correlated to the degree of frontal lobe hypometabolism [25]. One study by Cistaro et al. reports the degree of metabolism, with motor cortex hypometabolism in a range of 4-5 standard deviations compared to controls, midbrain and brainstem hypermetabolism in a range of 3-5 standard deviations compared to controls, and medial temporal hypermetabolism in range of 3-5 standard deviations compared to controls [29]. The magnitude of abnormality on PET can be dependent on normalization methods, image acquisition protocols, and reconstruction techniques, and for clinical use of PET with respect to discriminatory ability, the pattern of hypometabolism may be more critical than the magnitude of change. Currently, the anatomic distribution is emphasized in the clinical recognition of other neurodegenerative processes rather than the numerical thresholds and quantitative measurements which are most useful for identifying patters of abnormalities in group-level comparisons [35].

There are limitations to the diagram above. One is that several ALS phenotypes are pooled at once. Each phenotype may be distinct in its own way, but the results across all ALS phenotypes and studies have been condensed into the figure. Some regions may be overrepresented by a particular phenotype, meaning that the region may not be common to most ALS patients. Nevertheless, there are still common trends that emerge, namely, the frontal and motor hypometabolism and cerebellar and brainstem hypermetabolism.

Some regions of the brain have variable results concerning hypermetabolism and hypometabolism, particularly the temporal, occipital, and parietal lobes. In the temporal lobe, hypometabolic regions identified include Heschl's gyrus [26, 27] and the temporal cortex itself [20, 22, 33], while hypermetabolic findings include the temporal pole [18, 27] and medial temporal cortex [19, 24, 25]. In the occipital lobe/cortex, studies have identified it both as hypometabolic [20, 33, 34] and hypermetabolic [21, 25, 32]. The parietal lobe appears to have a slight hypometabolic bias, as three studies have found hypometabolism (inferolateral parietal cortex, left inferior parietal lobule, and the parietal cortex as a whole) [19, 28, 33] but one study found parietal hypermetabolism in a number of regions (superior and inferior parietal gyri, angular gyrus, precuneus, and postcentral gyrus) [29]. Other specific regions with mixed results include the insula [18, 26, 27], fusiform gyrus [24, 27, 29], amygdala [18, 25, 29, 33], lingual gyrus [27, 29], primary visual cortex [19, 23, 29], and paracentral lobule [22, 24]. However, the distribution of hypometabolism and hypermetabolism is not equal in some of these regions. For example, in the amygdala, three of the studies report hypermetabolism [18, 25, 29] while the other does not.

A reason for mixed results could be that certain lobes or cortices of the brain might have their own metabolic signature, with specific hypermetabolic and hypometabolic regions. For example, the hippocampus has been found to be hypermetabolic in ALS. The hippocampus is a part of the temporal lobe, so at the very least, this could suggest that the temporal lobe contains some hypermetabolic regions, if not being hypermetabolic all together. In addition, heterogeneity of metabolism within brain regions may not be reflected in group-level analysis, leading to mixed results for some anatomic locations. Potentially, as neurodegeneration progresses in more advanced cases, distributed networks in the brain may alter metabolism in regions remote from the center of neurodegeneration. Variation in representation of disease severity between studies could potentially account for mixed results in certain anatomic regions. A final reason could be that different ALS phenotypes have slight variations in metabolism, thus leading to variation in regions. Several ALS phenotypes have been investigated in [^18^F]FDG studies, such as genetic variants, sALS, and ALS-FTD. A particular phenotype may be responsible for only hypometabolic results in one part of the brain, but that region may appear variable due to being compared to different phenotypes. Overall, more research is required before making definitive conclusions on these seemingly variable regions of the brain.

In addition to the discussion of variable regions above, there was a study by Liu et al. that appears to flip the commonly seen metabolic pattern in ALS. In this study, hypometabolism was found in the cerebellum and midbrain, while hypermetabolism was observed in the anterior cingulate and the prefrontal lobe [36]. This is a pattern much different from what other studies have found. A potential reason for this, which the authors acknowledge as a limitation, is that this study took place in China, whereas all the other metabolic studies discussed are in primarily European and American populations. This highlights the need for more ALS research in all parts of the world to prevent a gross generalization. If [^18^F]FDG is to be used clinically, it is imperative that patterns and variations are established in the regions of the world so clinicians are better equipped to deal with patients from all backgrounds.

#### 2.1.1. Metabolic Patterns in Individual ALS Phenotypes

While the above section discusses ALS metabolism as a whole, individual phenotypes of ALS may have slight variations from the overall pattern. In this section, we review the metabolic findings of each phenotype of ALS.

*(1) Metabolic Patterns in Spinal and Bulbar Onset of ALS*. A study by Cistaro et al. compared spinal- and bulbar-onset ALS to controls and to each other. In the spinal group, they found hypermetabolism in the right midbrain and pons while hypometabolism was found in the lingual gyrus and right fusiform gyrus [29]. Pagani et al. did a similar comparison using the spinal subgroup of their study and found hypermetabolism in the bilateral midbrain, superior temporal gyrus, and right cerebellum while hypometabolism was bilaterally found in the visual (primary and associative), prefrontal, and premotor cortices [23]. Sala et al. found hypermetabolism in the cerebellum and hypometabolism in the primary motor cortex in their spinal group [32]. A more recent study by Canosa et al. found frontal and occipital hypometabolism but no hypermetabolism [37].

These four studies also identified hypermetabolic and hypometabolic regions in bulbar ALS patients. Cistaro et al. found hypermetabolism only in the pons but noticed hypometabolism in a number of places, including the prefrontal and premotor cortices, right insula, anterior cingulate, precuneus, and inferior parietal lobe [29]. Pagani et al. found hypermetabolism in the midbrain only while finding hypometabolism in the motor, premotor, and prefrontal cortices [23]. Sala et al. found hypermetabolism in the occipital cortex and cerebellum while finding hypometabolism in the primary motor cortex [32]. The recent study by Canosa et al. showed frontal, temporal, and occipital hypometabolism, but like the spinal patients in this study, no hypermetabolism was observed [37]. Some general metabolic patterns are observed here, such as cerebellum and brainstem hypermetabolism and frontal and motor cortex hypometabolism, which line up with the overall trend in ALS.

*(2) Metabolic Patterns in ALS-FTD*. Hypermetabolism does not appear to be as common in ALS-FTD. Another one of Cistaro et al.'s studies found hypermetabolism compared to controls in only the left cerebellum and midbrain [18]. Interestingly, contrary to this study, Rajagopalan and Pioro have conducted additional studies with ALS-FTD patients, and only one study out of three found hypermetabolism [26, 27, 38]. In the study with hypermetabolism findings, the hypermetabolic regions were the cerebellum, temporal gyri, occipital gyri, precuneus, and other gyri (angular, lingual, and fusiform).

Hypometabolism in ALS-FTD, however, is prevalent across all three studies. Cistaro et al. provide the hypometabolic Brodmann areas of the brain (6, 8, 9, 44, 45, and 47), corresponding to frontal areas [18]. The three studies by Rajagopalan and Pioro detail several gyri as being hypometabolic. Among them, there is consensus on the precentral gyrus, superior frontal gyrus, middle frontal gyrus, and inferior frontal gyrus [26, 27, 38]. The insula also is hypometabolic in the studies although it is the right insula in one study [38] and the left in the other two [26, 27]. Individual studies list other hypometabolic regions such as the gyrus rectus [26, 27], anterior, medial, and paracingulate gyri [26, 27], and occipital and temporal areas [38].

Regions with mixed findings are the postcentral and supramarginal gyri and parietal areas (parietal gyri and parietal lobules), with them being hypermetabolic in one study [27] and hypometabolic in another [38]. Sample size is a limitation in these studies, as all four of these ALS-FTD studies had under 20 subjects.

The frequent copathology of ALS and FTD likely reflects a spectrum of underlying TDP-43 proteinopathy, implicated in both conditions. Of patients with FTD, approximately 15% meet ALS criteria, and a greater percentage have suggested clinical features of ALS [30]. The [^18^F]FDG PET regions of hypometabolism have significant overlap in the frontal lobes, likely reflecting the similarities between the conditions. Frequently in FTD, [^18^F]FDG uptake in the sensorimotor cortex is preserved whereas the primary motor cortex is a prominently reported region of hypometabolism in ALS [39]. In ALS-FTD, more extensive temporal hypometabolism has been reported [40]. While there is promising evidence for the use of [^18^F]FDG PET as a biomarker to support a diagnosis of ALS, the routine use for distinguishing ALS from other neurodegenerative conditions or for evaluating the cognitive impairment in ALS is not supported by clinical guideline recommendations or expert consensus [41].

*(3) Metabolic Patterns in C9ORF72 ALS*. In a study comparing C9ORF72 ALS to controls, hypermetabolism was found in the midbrain, cerebellum, hippocampus, amygdala, insula, and temporal pole [18]. Another study by Castelnovo et al. observed metabolic patterns in three C9ORF72 ALS patients. One patient had hypermetabolism in the vermis and cerebellar cortex while the other two had widespread cortical and subcortical hypermetabolism [42].

With regard to hypometabolism, Cistaro et al. identified the bilateral caudate, thalamus, insula, and several frontal cortex regions [18]. The three patients in Castelnovo et al.'s study had variable hypometabolism, but regions that frequently came up were the thalamus, inferior frontal gyrus, and postcentral gyrus (each appeared in two of the three patients) [42]. Van Laere et al.'s study described a pattern of C9ORF72 hypometabolism that was similar to that of ALS patients compared to controls (where the ALS pattern of hypometabolism was in the prefrontal, lateral frontal, and premotor cortex), with a slight emphasis on the thalamus, posterior cingulate, and precuneus [25]. If more research is done to identify regions specific to C9ORF72 ALS or any other genetic variant, then the regions could be used to aid diagnosis in patients who have a C9ORF72 mutation (or other gene mutant) that puts them at risk for developing ALS.

*(4) Metabolic Patterns in TARDBP ALS*. There is not much literature on brain metabolism in TARDBP ALS. Canosa et al. analyzed TARDBP-mutated ALS cases with [^18^F]FDG. In the study, they found widely distributed hypometabolism bilaterally in frontal, parietal, temporal, and occipital regions compared to healthy controls [20]. More research will need to be done on TARDBP ALS to determine if a similar metabolic pattern occurs.

*(5) Metabolic Patterns in SOD1 ALS*. Similar to TARDBP ALS, there is a literature deficit in SOD1 ALS brain metabolism. In contrast to their TARDBP ALS study, Canosa et al. found no hypometabolism in SOD1 ALS patients compared to controls. The hypermetabolism was in the frontal and parietal lobes (Brodmann areas 4-6 and 40) [22]. This finding, most notably the frontal hypermetabolism, is in contrast to several ALS studies that have consistently found frontal hypometabolism. This finding would need additional studies to confirm it, but if it is shown to be consistent in SOD1 ALS, it could be a valuable biomarker in diagnosis in SOD1 mutated patients.

#### 2.1.2. Comparisons of Glucose Metabolism among Certain ALS Phenotypes

Certain brain regions have had hypermetabolism or hypometabolism in ALS across several phenotypes, but some phenotypes might have a more severe metabolic pattern than another. For example, two phenotypes may both exhibit frontal hypometabolism, but that does not necessarily imply that the extent or intensity of that hypometabolism is the same. The purpose of this section is to review the metabolic comparisons across ALS subgroups that have been done. These results are summarized in Tables 1 and 2.

#### 2.1.3. Additional Applications of Metabolic Imaging

While [^18^F]FDG is used for assessing metabolism, there are additional uses of it than just determining metabolic patterns in ALS and its phenotypes. Here, we discuss many of the other uses of [^18^F]FDG data.

**Figure 3:**
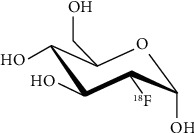
The structure of [^18^F]FDG, which has uncovered a specific signature of glucose hypermetabolism and hypometabolism in ALS, along with being applied to a number of other purposes [18–34, 36–38, 42–47].

*(1) As a Discriminatory Marker*. Certain studies have assessed [^18^F]FDG-PET (Figure 3) as a discriminatory tool, trying to see if models can successfully distinguish ALS from either controls or related syndromes.

Studies trying to distinguish ALS cases from controls have generally found high accuracy. Van Laere et al.'s study found an accuracy of 94.4% using a volume of interest (VOI) analysis to distinguish strictly ALS from controls. Additionally, a support vector machine (SVM) analysis done on the same groups (cases and controls) showed sensitivity, specificity, and accuracy of 94.8%, 80.0%, and 91.8%, respectively. When narrowing the ALS group down to C9ORF72 ALS, sensitivity, specificity, and accuracy were 90.9%, 100%, and 96.8%, respectively [25]. Van Weehaeghe et al. divided ALS cases into two groups, where metabolic patterns in the first group were used for discriminatory purposes in the second. Using this method resulted in sensitivity, specificity, and accuracy of 88.6%, 90.0%, and 88.8%, respectively, in the second ALS group compared to controls through VOI analysis. SVM analysis yielded 100% sensitivity, specificity, and accuracy in the second group. When both ALS groups were pooled together against controls, sensitivity was 100% while specificity and accuracy were both 95% [19].

Sala et al.'s study on spinal- and bulbar-onset ALS found poor results when it came to discriminating spinal and bulbar onsets. A discriminant analysis was only able to correctly classify 51.43% of bulbar cases and 65% of spinal cases, while their best SVM approach was able to correctly classify 34.28% of bulbar cases and 85% of spinal cases [32]. In addition to these comparisons, some studies evaluated if [^18^F]FDG could distinguish ALS from ALS-like conditions, such as mimics like PLS and PMA. Another study by Van Weehaeghe et al. found classifying accuracies of 60.6 ± 2.3% for ALS versus ALS mimics including PLS and 59.2 ± 3.7% for ALS mimics including PMA. Predictive accuracy was slightly improved when based on regions of the brain that showed significant differences between the groups [47]. Van Laere et al.'s study additionally did classifications with PLS and PMA. They found an accuracy of 89.7% in a group of ALS, PLS, and controls, although this figure dropped to 62.4% when adding PMA cases to the group [25]. Overall, these results show that [^18^F]FDG is an excellent marker to distinguish ALS from control cases, but it may have some limitations when distinguishing ALS phenotypes or ALS from similar conditions.

*(2) ALS Staging*. A study used [^18^F]FDG to track brain metabolism through disease progression. Disease progression was tracked by King's stages, a recent ALS staging system [44]. The stages are correlated with ALS's effects on the body, where more parts of the body are affected in later stages. In stage 1, the first region of the body is affected by ALS as symptoms begin. Stage 2A is diagnosis of ALS and 2B involves a second region being affected by ALS. Stage 3 adds a third region. Stage 4 involves medical interventions, where 4A is gastrostomy and 4B is noninvasive ventilation. In this study, patients ranged from stages 1 to 3 (only involvement of a second region was counted for stage 2), and metabolism was compared among the stages. Researchers found no significant differences in metabolism between stages 1 and 2. They pooled stages 1 and 2 together and compared them to stage three. The stage three group showed hypometabolism compared to stages 1 and 2 in several places, including Brodmann areas 3, 4, 6, 8, and 9 in the right hemisphere (corresponding to the frontal and parietal cortices) and areas 4 and 6 in the left hemisphere (frontal) [44]. These results indicate spreading of hypometabolism as ALS worsens, suggesting that hypometabolism may originate in a particular region of the brain and expand from there. However, given the cross-sectional design of the study, a longitudinal study using [^18^F]FDG and King's staging system could further confirm this finding. Such a study would be able to confirm the worsening disease progression within a patient instead of relying upon comparisons between patients.

*(3) Correlation with Apathy*. Apathy is a lack of emotion and motivation that has been observed as a comorbidity in various neurological disorders, including ALS. Using the Frontal Systems Behavior Scale (FrSBe), apathy can be assessed. The FrSBe has a “before” and “after” score, referring to two time points at which the test is taken, where “before” refers to prediagnosis and “after” is at diagnosis. A higher score in general indicates a worsened apathy condition. In one study, FrSBe scores of ALS patients were compared to brain metabolism [45]. The after score negatively correlated with metabolism in anterior cingulate cortex, premotor cortex, insula, and parts of the prefrontal cortex, while positively correlating with the cerebellum and pons. A very similar correlation occurred with the difference in before-after scores as compared with metabolism. Given that several of the negatively correlating brain regions are known to be hypometabolic in ALS (and the positively correlated brain regions are hypermetabolic), it seems that apathy correlates with the metabolic pattern seen in ALS, where worsened hypermetabolism and hypometabolism are associated with worsened apathy. Additional research on apathy and brain metabolism can be done to observe if apathy can be used to predict metabolic patterns indicative of ALS.

*(4) As a Predictor of Survival*. A study attempted to correlate [^18^F]FDG images in patients to survival time. Particular brain regions were identified and used for prediction through an SVM after the SVM was trained using data from some of the ALS patients. Three survival classes, 0-2 years, 2-5 years, and >5 years, were the survival times used for SVM training and prediction. The classification error rates in these classes were found to be 19.51%, 24.39%, and 4.87% [46]. Imaging as a tool for prognosis would help clinicians determine the next steps for care in an ALS patient, along with allowing patients and their families to have an idea of the progression of the disease. However, more studies of this nature would need to be done to ensure that such analysis can apply to other populations. In addition, further research can be done to increase the accuracy of classification.

### 2.2. Neuroinflammation Imaging of ALS

**Figure 4:**
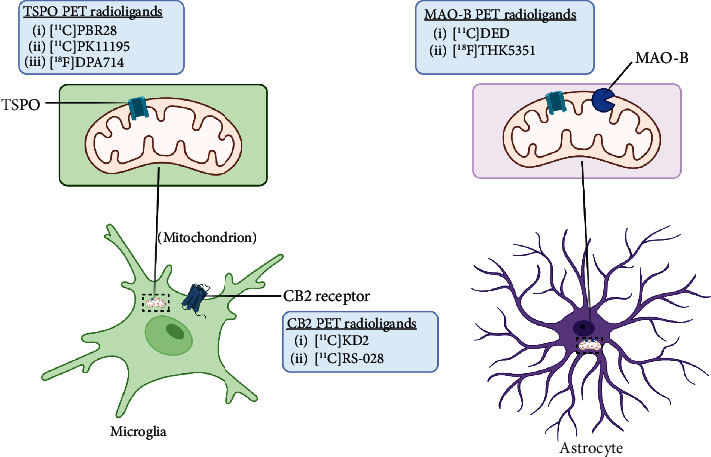
A summary of neuroinflammation targets, their location, and respective PET ligands developed in ALS.

Neuroinflammation is a protective response against the neuronal injury caused by either traumatic brain injury, toxicity, or infection. During this process of inflammation, glial cells, which are innate resident nonneuronal cells, get activated and trigger various biochemical responses to protect neuronal cells. During this process of neuroinflammation, various biochemical pathways also get activated, including secretion of cytokines and chemokines to target the migration of microglial cells, expression of translocator protein on mitochondria of activated glial cells, expression of various enzymes (monoamine oxidases) on mitochondria of astrocytes, and expression of cannabinoid type 2 receptors (CB2R) on microglia. Several PET radiotracers have been developed to track neuroinflammation. Studies using such radiotracers give insight into the role and extent of neuroinflammation in ALS. A summary of the radiotracers and the location of their targets is provided in Figure 4 at the end of this section.

#### 2.2.1. Imaging of Translocator Protein in ALS

Over the years, various PET probes have been developed to estimate the presence of 18-kilodalton translocator protein (TSPO) expressed on the mitochondria of the activated glia, such as microglia and astrocytes [48], to assess the degree of neuroinflammation in ALS patients (Table 3).

*(1) [^11^C]PBR28*. In a longitudinal study with a transgenic mouse model (SOD1^G93A^ mutation), [^11^C]PBR28 binding kept increasing, indicating the progressive development of neuroinflammation. Increased binding was found throughout the body, including in the lungs, spinal cord, hindbrain, and brainstem [49]. One of the studies in ALS patients with [^11^C]PBR28 found an increased uptake in the precentral gyrus, corticospinal tract, and several motor areas in patients as compared to controls [50]. Another study found similar results, showing increased uptake in the motor cortices through voxel analysis and increased uptake in the precentral and paracentral gyri using surface-based analysis in ALS patients compared to controls. However, the researchers added primary lateral sclerosis patients to this study as well and found that ALS exhibited no increased uptake compared to PLS, while PLS had increased uptake in the white matter of the motor cortices in the voxel-wise analysis [51]. In addition to these two studies, another study showed increased uptake in the primary motor cortices [52].

Some studies have observed clinical correlations between [^11^C]PBR28 uptake and certain clinical markers of ALS severity. One study found a positive correlation between [^11^C]PBR28 uptake in the bilateral precentral gyri and the upper motor neuron burden (UMNB), which assesses the severity of upper motor neuron signs. This study also found no association between [^11^C]PBR28 uptake and ALSFRS-R scores [53]. Another study tried to correlate [^11^C]PBR28 uptake with two different upper motor neuron burden scales (UMNS): the Penn UMNS and the MGH (Massachusetts General Hospital) UMNS. The Penn UMNS scale is more comprehensive and covers more symptoms of UMN degeneration than the MGH UMNS. This study saw a positive correlation with primary motor cortex [^11^C]PBR28 binding and scores in both scales [54]. The study by Zürcher et al. found correlations between the uptake from 60 to 90 minutes (“SUV_60-90_ min”) and many measures, such as a positive correlation with UMNB score and a negative correlation with ALSFRS-R score [50]. The study by Alshikho et al., using [^11^C]PBR28 uptake in the white and gray matter, found a negative correlation with the fine motor domain of the ALSFRS-R and a positive correlation with the UMNB [51]. These results suggest that [^11^C]PBR28 can be used as a marker of UMN degeneration.

*(2) [^11^C]PK11195*. [^11^C]PK11195 is another small molecule that binds TSPO and can be used to measure its overexpression during glial activation in ALS. Turner et al.'s early study with [^11^C]PK11195 found significantly increased binding in the motor cortex, pons, frontal lobe, and thalamus in sALS patients compared to controls. This study also found a positive correlation between the binding potential of [^11^C]PK11195 in these regions (notably the thalamus and motor cortex) and their upper motor neuron score. No correlation was seen with ALSFRS-R [55]. A more recent study by Tondo et al. measured [^11^C]PK11195 uptake in symptomatic and asymptomatic SOD1-mutated carriers and controls. When compared to controls, symptomatic patients had increased microglial activation in the occipital, temporal, premotor frontal, and parietal cortices, thalamus, cerebellum, and medulla. Asymptomatic patients had increased microglial activation in the occipital, temporal, premotor frontal, supplementary motor, and parietal cortices and cerebellum compared to controls [56].

*(3) [^18^F]DPA714*. [^18^F]DPA714 is a more recent TSPO target. One study that evaluated [^11^C]PBR28 also used [^18^F]DPA714 on a group of ALS patients, comparing them to controls. Like [^11^C]PBR28, [^18^F]DPA714 uptake was significantly increased in the primary motor cortices of ALS patients [52]. In addition to this clinical study, one study applied [^18^F]DPA714 to SOD1^G93A^ mouse models. This study found increased uptake in the brainstem, cerebellum, and cervical spinal cord in the SOD1^G93A^ mutant mice compared to wild-type mice, but the brainstem was the only significant region [57]. There have been challenges in using older TSPO targeting tracers, such as issues with noise and uptake [58]; hence, development on newer tracers like [^18^F]DPA714 may prove useful in better quantifying neuroinflammation.

#### 2.2.2. Imaging of Monoamine Oxidase B (MAO-B) in ALS

Monoamine oxidase B (MAO-B) is an enzyme present on the membrane of astrocyte mitochondria [59, 60]. Two PET ligands targeting MAO-B have been applied in the study of ALS pathology. One radioligand is deuterium-substituted [^11^C](l)-deprenyl, also known as [^11^C](l)-deprenyl-D2 or [^11^C]DED. In one study, an increased rate of uptake in ALS patients compared to controls was found in the pons and in white matter. Additionally, there was decreased uptake in the parietal and temporal cortices [59]. A radiotracer initially developed as a first-generation tau PET agent [^18^F]THK5351 has significant off-target binding to MAO-B [61]. Case reports of [^18^F]THK5351 in ALS may reflect a role of MAO-B. One case report on a patient with ALS and Alzheimer's disease found that [^18^F]THK5351 had increased uptake in the precentral gyri, medial temporal lobe, and inferior temporal lobe after comparison with uptake images of controls [60]. Another report on two ALS patients (with control reference images as well) found increased uptake in the motor cortex of one patient, while in the other, increased uptake was seen in the left anterior temporal lobe [62]. These two reports, totaling only three patients, do not provide enough information to conclusively determine which areas have elevated [^18^F]THK5351 uptake. In short, additional studies with a larger sample size would need to be conducted with [^18^F]THK5351 to determine its value as a marker of astrocytic neuroinflammation. Another limitation to using MAO-B for inflammation imaging is the physiologic expression in neurons, as the primary role for this enzyme is the degradation of monoamine neurotransmitters.

#### 2.2.3. Imaging of Cannabinoid Receptor Type in ALS

Cannabinoid receptor type 2 (CB2R) is a G-protein-coupled receptor. In several neurological disorders, increased expression of CB2R on activated microglia has been observed [63]. There have not yet been clinical studies evaluating the in vivo effectiveness of CB2R tracers, but there have been autoradiography studies done on human tissue. One study developed [^11^C]KD2 to bind to CB2R. [^11^C]KD2 autoradiography was done on postmortem ALS spinal cord tissue with and without a CB2R agonist. There was qualitatively higher uptake in the ALS tissue without the CB2R agonist, demonstrating that [^11^C]KD2 was selectively binding to the CB2 receptors [64]. However, there was no control tissue in the study, so increased CB2R in ALS was not demonstrated. Another study using [^18^F]RS-126 did autoradiography with postmortem ALS spinal tissue compared to healthy tissue. This time, there was higher uptake in the ALS tissue compared to the control tissue as seen by the greater intensity in the autoradiography image. However, specific binding was not seen when a CB2R ligand was applied to the ALS tissue. [^18^F]RS-126 uptake did not drop, meaning it was likely binding to other targets. The researchers in this study decided to do autoradiography with a similar tracer, [^11^C]RS-028. Using the same setup, significantly higher [^11^C]RS-028 uptake was seen in ALS postmortem spinal tissue compared to control tissue and ALS tissue with a CB2R ligand, showing specific and increased binding [63].

Overall, these studies show that [^11^C]RS-028 and [^11^C]KD2 may be useful as a PET tracer to selectively observe CB2R expression. Future studies can be done with ALS patients against controls to further determine its utility in detecting neuroinflammation.

### 2.3. Synaptic or Neuronal Density Imaging of ALS

Ionotropic neurotransmitter receptors are present on the neuronal and glial cells. These neurotransmitter receptors are responsible for sending messages from one neuron to another at a synapse. There are different classes of neurotransmitter receptors such as adrenergic, cholinergic, dopaminergic, GABAergic, glutamatergic, and serotonergic. Several radiotracers have been developed with the sole purpose of binding to these neuronal receptors which can be applied to evaluate their density in ALS compared to controls (Table 4).

#### 2.3.1. [^11^C]flumazenil

[^11^C]flumazenil is a radiotracer that acts as a marker for neuronal density by binding to the GABA_A_ receptors of neurons, specifically on the benzodiazepine subunit [65]. The earliest study with [^11^C]flumazenil in ALS assessed uptake through “flumazenil volumes of distribution” (FMZVD). Several decreases in FMZVD compared to controls were identified in parts of the brain, such as Brodmann areas 7, 9, 10, 18 (bilaterally), 19, 21, (right hemisphere), 4, and 45-47 (left hemisphere). These represent mainly frontal and motor areas although other regions from different lobes are represented [66]. Another study later found comparable results, with sALS patients having decreased binding in premotor and motor regions as compared to controls. However, this study included a cohort of ALS patients homozygous for a D90A mutation on the SOD1 gene (“homD90A”). The study found decreased [^11^C]flumazenil binding in the frontal lobe (including the left frontotemporal junction) and anterior cingulate gyrus compared with controls. Additionally, a few clinical correlations were identified in the homD90A group, with decreased left frontotemporal junction binding correlating with disease duration and decreased binding in several frontal regions correlating with the ALSFRS-R score. A negative correlation between decreased binding and UMN score was found in the sALS group [6].

Finally, a study comparing neuropsychological assessment in ALS patients against [^11^C]flumazenil binding found that decreased binding in the right inferior frontal gyrus, superior temporal gyrus, and anterior insula was correlated with worsened performance on the Written Verbal Fluency Test, where patients were required to generate certain words and later copy them down. Another correlation was found between poorer scores on the Graded Naming Test (GNT) and decreasing binding in the left cuneus and an area between the left inferior and middle frontal gyri. The GNT was a test where patients were made to name objects from drawings [65].

#### 2.3.2. [^11^C]WAY100635

[^11^C]WAY100635 is a ligand developed for binding to the 5-hydroxytryptamine receptor (5-HT_1A_), a serotonin receptor found on many neurons throughout the nervous system. A study using [^11^C]WAY100635 in ALS demonstrated overall reductions in binding potential in patients compared to controls. Through VOI analysis, volumes of interest with large decreases included several gyri, such as the fusiform, parahippocampal, medial inferior temporal, precentral, intermediate frontal, and orbitofrontal gyri. Statistical parametric mapping (SPM) analysis showed binding decreases in frontotemporal regions, cingulate, and precentral gyri [67]. There were no significant clinical correlations, as any correlations identified did not make it past statistical mutiple comparison corrections. The authors postulated that the decreased binding could reflect a decreased amount of the 5-HT_1A_ receptor, fewer neurons expressing the 5-HT_1A_ receptor, or simply a lessened affinity of [^11^C]WAY100635 for 5-HT_1A_.

#### 2.3.3. Metabotropic Glutamate Receptor Subtype 5 Tracers

[^18^F]FPEB is a ligand for metabotropic glutamate receptor subtype 5 (mGluR5). Being glutamate receptors, mGluR5 levels can be used to assess glutamatergic activity, as hyperactive glutamate function can contribute to neurotoxicity. In a study with [^18^F]FPEB, SOD1^G93A^ mouse models showed higher binding potentials of the tracer as compared to control mice in the overall brain. The hippocampus was the region with the biggest difference between ALS model and control mice, although other regions included the striatum and frontal cortex. In addition to this result, the mouse models were tracked across ALS stages, with binding potential increasing from stage one to three. These results indicate higher mGluR5 expression levels in the ALS model mice, which only increase as the disease progresses. The higher expressions of mGluR5 can help explain the mechanism of glutamate mediated excitotoxicity, since excessive activation can lead to neuron death [49].

Another study assessed another tracer, [^18^F]PSS232, in neuroinflammation-induced mice and ALS postmortem brain tissue. Mice had induced neuroinflammation through a lipopolysaccharide (LPS) injection, while control mice had a “vehicle” injection that was just a NaCl solution. In the mice, higher [^18^F]PSS232 binding was found in the C57BL/6 neuroinflammation induced mice in several regions of the brain, such as the striatum, hippocampus, and cortex a day after injection. In the autoradiography experiment, the ALS tissue exhibited significantly higher binding of [^18^F]PSS232 than in control non-ALS postmortem brain tissue, namely, the basal ganglia, and the frontal, motor, and temporal cortices [48].

#### 2.3.4. [^18^F]fallypride

[^18^F]fallypride is an antagonist radiotracer for D_2_ and D_3_ dopamine receptors. In a study utilizing this tracer, lower uptakes were found in the superior frontal gyrus, nucleus accumbens, left temporal lobe, and angular gyrus in ALS patients compared to controls. The patients and controls underwent neuropsychological assessment, where patients scored worse on language and delayed recall components of the Montreal Cognitive Assessment (MoCA). The authors correlated this poorer performance to the decrease in binding to dopamine receptors, suggesting that the number of dopamine receptors decreases in ALS, even before symptoms appear, indicating that ALS disrupts dopaminergic function [68].

#### 2.3.5. [^18^F]SynVesT-1

[^18^F]SynVesT-1 is a PET radiotracer for synaptic vesicle glycoprotein 2A, a vesicular membrane protein found on synapses. A study comparing ALS patients to controls showed uptake decreases in the inferior frontal gyrus, anterior cingulate, hippocampus-insula region, and right temporal lobe. This study also compared spinal-onset patients to bulbar-onset patients. Bulbar-onset patients had lower uptake in the cingulate gyrus but higher uptake in the superior temporal gyrus and left occipital lobe. The different onsets were also compared to controls. Bulbar-onset patients had lower uptake in the temporal lobe, occipital lobe, and insula compared to the controls. Interestingly, spinal-onset patients had no significant uptake differences compared to controls. In addition, the study compared cognitively impaired ALS to cognitively normal ALS and controls. No significant differences were seen between the two ALS groups. When compared to the controls, cognitively impaired ALS had lower uptake in the superior temporal gyrus, anterior cingulate, hippocampus-insula region, and left inferior frontal gyrus [69].

#### 2.3.6. [^18^F]OF-NB1

[^18^F]OF-NB1 is a tracer developed for N-methyl-D-aspartate receptor type 2B. A study was done with this tracer, giving it to rats to first assess binding selectivity and then to postmortem ALS tissue (autoradiography). In the autoradiography, brain tissue from Brodmann areas 4 and 6 (from motor and frontal cortices) were used, where significantly less binding was found compared to control brain tissue. These results were confirmed with immunohistochemistry [70].

### 2.4. Pathological Protein Imaging

This category of imaging is different from the previous three as this one quantifies aggregate formation in ALS, while the others targeted metabolism, neuroinflammation, and synaptic density. At present, there is very limited research on imaging such aggregates.

#### 2.4.1. TDP-43 Imaging of ALS

Various efforts have been made to develop a selective PET probe to specifically target and image the TDP-43 protein aggregates noninvasively, but it remains a daunting task. Additionally, researchers have attempted to explore the possibility of repurposing the existing PET probes (from their respective fluorinated versions) to specifically image TDP-43 using PET. In this study, tritium analogs of six radiotracers ([^3^H]MK-6240, [^3^H]Genentech Tau Probe-1, [^3^H]JNJ-64326067, [^3^H]CBD-2115, [^3^H]flortaucipir, and [^3^H]APN-1607) were tested for in vitro binding with postmortem brain tissues of ALS to evaluate their imaging potential. Unfortunately, none of these six tritiated analogs of radiotracers demonstrated specific binding to phosphorylated TDP-43, as some of the radiotracers had marginal binding, or attached to non-TDP-43 targets [71]. As of now, PET imaging of TDP-43 remains an unmet clinical need. Direct imaging of not only TDP-43 protein aggregates but those stemming from other genes as well may allow quantitative visualization of such aggregates in patients. Future radiotracer development may enable protein aggregate visualization through PET to aid in staging or assessing progression of ALS.

#### 2.4.2. Amyloid Imaging

Cerebrospinal fluid analysis has suggested a potential link between amyloid pathology and ALS [72]. One of the [^18^F]FDG studies also used [^18^F]florbetaben to image amyloid protein in ALS patients. In the study, no significant difference in uptake was found between patients and controls, although some individual patients had increased uptake of [^18^F]florbetaben [28]. At present, no strong link between amyloid plaques and ALS has been found in imaging studies, but given the paucity of literature on this topic, further studies with [^18^F]florbetaben or other amyloid radiotracers may provide more information about the role of amyloid in ALS.

### 2.5. Single-Photon Emission Computed Tomography

Single-photon emission computed tomography (SPECT), like PET, is another imaging modality that acquires cross-sectional imaging of the distribution of an injected radiotracer. However, PET detects two coincident 511 keV photons from positron annihilation, while SPECT detects single gamma ray emissions from radiotracers within an energy range of 50-400 keV. It should be noted that the costs of SPECT infrastructure and radiotracers are generally far less than PET, making the former more accessible across the globe. Application of SPECT imaging in ALS is summarized below with respect to each radiotracer.

#### 2.5.1. [^123^I]-Labeled Radiopharmaceuticals

One study utilized [^123^I]IPT [N-(3-iodopropen-2-yl)-2*β*-carbomethoxy-3*β*-(4-chlorophenyl)tropane] to determine dopamine transporter levels in ALS patients and controls, finding significantly decreased striatal radiotracer uptake in ALS involving the caudate and putamen. Between patients with bulbar- and spinal-onset cases, there were not any significant differences observed as both showed similar decreases in binding [73]. This study appears to agree with the results of the PET study with [^18^F]fallypride [68], adding evidence to dopaminergic dysfunction in ALS. Another study used ^123^I-labeled isopropyl amphetamine ([^123^I]IMP) with the goal of measuring the regional cerebral blood flow (rCBF) in ALS patients. In addition to a control group, there were two groups of ALS patients in this study: ALS with dementia (ALS-D) and ALS without dementia. In the ALS-D group, there were regions of decreased rCBF in the frontal lobe, particularly the premotor region, compared to the ALS-only group and controls. When comparing the ALS-only group to controls, there were rCBF decreases, although not as drastic as the ALS-D group, in the anterior cingulate gyrus and posterior corpus callosum [74].

#### 2.5.2. [^99m^Tc]-Labeled Radiopharmaceuticals

[^99m^Tc]Tc-ethyl cysteinate dimer or [^99m^Tc]Tc-ECD and [^99m^Tc]Tc-hexamethyl propylamine oxime (HMPAO) are two SPECT radiotracers frequently used to assess cerebral perfusion. A study using [^99m^Tc]Tc-ECD found positive correlations between the ALSFRS-R score and [^99m^Tc]Tc-ECD perfusion of patients in multiple regions of the right hemisphere, including the insular cortex and parts of the frontal lobe (Brodmann areas 6, 8, and 44). Within the ALSFRS-R, positive correlations were found between the bulbar score and perfusion in the right insular cortex, frontal lobe (middle and inferior frontal gyri), and in the left precentral gyrus. Regarding the lower limb score in the ALSFRS-R, positive correlations were seen in several regions in the left and right hemispheres. Correlated regions in both hemispheres included the superior, middle, and precentral gyri, while the inferior parietal lobule was a left hemisphere-specific area, and the postcentral gyrus and anterior cingulate were right hemisphere-specific [75]. Another study applied [^99m^Tc]Tc-ECD SPECT to patients with ALS-PDC (parkinsonism-dementia complex) in the Kii Peninsula of Japan, a hotspot for this condition. Regional cerebral blood flow was decreased in the frontal and temporal lobes of patients [76]. Finally, one study performed [^99m^Tc]Tc-ECD SPECT on limb-onset ALS and primary bulbar palsy (PBP) ALS. A comparison was made between the rCBFs of both groups, finding that the ALS-PBP patients had a much lower rCBF in the frontal lobe compared to the limb-onset patients. In addition to this group comparison, rCBFs in brain regions within each patient group were compared. In the limb-onset group, frontal rCBF was lower than several other regions (parietal, occipital, temporal lobes, brain stem, and cerebellum). A similar trend with the frontal lobe was observed in the ALS-PBP group, but as an additional comparison, the anterior cingulate gyrus had a lower rCBF than the motor cortex [77].

Studies using [^99m^Tc]Tc-HMPAO are not as recent as most studies with [^99m^Tc]Tc-ECD, with many performed in the 1990s, likely reflecting trends in institutional choices of perfusion agents. One of the earliest studies sought to determine how rCBF was affected in ALS patients and measured the global rCBF in patients and controls through [^99m^Tc]Tc-HMPAO SPECT and [^133^Xe] inhalation. [^133^Xe] inhalation was unrevealing, but [^99m^Tc]Tc-HMPAO SPECT demonstrated a significant decrease in rCBF. When comparing individual regions in the brain, the frontal lobe and hippocampus had greater rCBF decreases overall, although some patients had rCBF decreases in other regions as well [78]. Other studies have followed suit, determining where such perfusion decreases exist. A study of five ALS-FTD cases found decreased perfusion in frontal and temporal regions, similar to the other [^99m^Tc]Tc-HMPAO study [79]. In addition to the previous two studies, another divided ALS patients into three groups based on performance on certain cognitive assessments. Group one had the lowest score on the Mini Mental State Examination, where groups two and three had increasing scores (three had the highest). Groups one and two in this study exhibited low uptake in the frontal region whereas group three had lower uptake in the rolandic (central sulcus) region [80].

## 3. Conclusions

Both PET and SPECT imaging have elucidated much about the mechanisms of ALS pathophysiology and highlighted various biochemical changes occurring at the molecular level in ALS patients. [^18^F]FDG is one of the most common radiotracers and has revealed a distinct pattern of glucose metabolism, with certain regions being hypermetabolic or hypometabolic. However, there still exist limitations to these studies. Most studies occur in primarily western populations and consequently overrepresent certain parts of the world. As seen by the [^18^F]FDG study in China, there may be slight metabolic differences in ALS from place to place. As a result, more studies should be performed in different populations for a better understanding of ALS metabolism. Another limitation is the lack of longitudinal studies. Studies have assessed the metabolic status of patients at once, but research is lacking on tracking metabolic patterns in patients throughout time. Such studies would take more time and resources but could reveal additional information about the progression of ALS, such as how a particular brain region may change metabolically from onset to death. In addition to the limitations described, there still exists some variability between studies on certain regions of the brain, particularly in temporal, occipital, and parietal areas. Such variability could have arisen from combining certain phenotypes of ALS, each with their own distinct signature. Other reasons could be due to limited sample sizes of studies or certain parts of the brain having their own variable metabolism within specific regions. Future [^18^F]FDG PET studies may classify ALS patients into their respective subtype, whether it be bulbar or spinal onset, ALS-FTD, or other phenotypes. Additional validation of the reported PET biomarkers is needed in prospective studies to demonstrate the clinical utility of these promising applications. At present, no clinical guidelines recommend the routine use of FDG PET in ALS. In future work, more detailed characterization and classification of ALS with PET could help in identification of a particular region of the brain with hypermetabolic or hypometabolic activity in specific ALS types, with the possibility to better target therapies for different subtypes and inform about the pathophysiology of ALS.

In addition to the unique metabolic patterns [^18^F]FDG has uncovered, there may be other uses for it in the future. There is value in using it as a discriminatory marker, especially when determining if there is presence of ALS, although such success is not seen when trying to distinguish ALS from similar conditions. More research and development would need to be done to determine if [^18^F]FDG can be used clinically to distinguish ALS from other similar conditions. Such a development could reduce the time required for a differential diagnosis, potentially allowing patients to receive treatment earlier, even as there is no cure to reverse ALS. In addition, a study has attempted to use [^18^F]FDG as a survival predictor. If enough research is done to translate [^18^F]FDG metabolic findings into clinical prognostic data, this would aid physicians in treating and managing ALS.

With respect to the neuroinflammation radiotracers, they generally have shown increased uptakes, indicating the increased presence of neuroinflammation in ALS. More studies can be done with several recently developed neuroinflammation radiotracers to compare their performance with preexisting radiotracers. There are already many well-known neuroinflammation targets, so new radiotracers can be synthesized to target any of them. The newer radiotracers have been utilized on ALS tissue, but further expansion to ALS patients and controls allow for a stronger comparison of neuroinflammation. In addition to clinical comparison studies, longitudinal studies tracking neuroinflammation over time can be performed to determine its temporality with respect to symptom onset.

With respect to neuronal density radiotracers, most, but not all, have demonstrated decreases in binding. These decreases represent neuronal death in ALS, characteristic of the motor neuron degeneration that is seen in ALS. Several different targets exist for this class of radiotracers, particularly receptors on neurons. By specifically targeting certain receptors, the presence of a certain type of neuron associated with it can be determined in ALS. Future advancements in neuronal targeting PET probes have the potential to uncover the several types of neurons affected, contributing to a better understanding of how ALS affects the nervous system. As already demonstrated, ALS interferes with dopaminergic, glutaminergic, and serotonergic functions.

There are lack of studies imaging the aggregation of harmful proteins in ALS. As of this review, there are studies on TDP-43 and amyloid protein formations. Future research can attempt to develop radiotracers for in vivo quantification of other involved proteins, like those from SOD1 and C9ORF72 mutations. Longitudinal studies with such tracers would allow researchers to correlate ALS onset to aggregate development.

While there are many different individual radiotracers for PET, not as many are present for SPECT. SPECT has demonstrated general decreases in rCBF in studies conducted on patients. However, there are simply less studies done with SPECT compared to PET. More research could be put into SPECT tracer development given its reduced cost and increased accessibility. As with other types of neuropathology, SPECT has great advantages for assessment of cerebral perfusion due to accessibility of radiotracers with high first-pass extraction. SPECT is also optimal for assessment of binary, focused decisions such as whether or not striatal uptake is diminished, analogous to the way [^123^I]ioflupane is used for Parkinson's disease or to the investigational use of [^123^I]IPT in ALS. Future work in SPECT radiopharmaceuticals could reveal diagnostic utility for ALS. Given the significant limitations of SPECT compared to PET in regard to spatial resolution, image contrast, and versatility of PET radiopharmaceuticals for imaging a variety of biochemical and molecular processes, future nuclear medicine research in ALS will likely focus on further realizing the potential of PET. These studies may explore future applications for existing radiopharmaceuticals in addition to novel radiopharmaceutical development. Results seen in these studies overall have and will continue to shed light on the pathophysiology of ALS.

## Figures and Tables

**Table 1:** Comparisons of commonly known ALS phenotypes. A dash indicates that no comparison has been done between the phenotypes.

ALS phenotypes	C9ORF72 ALS	ALS-FTD	sALS	SOD1 ALS
C9ORF72 ALS	—	—	—	—

ALS-FTD	Cistaro et al.'s C9ORF72 study found (in C9ORF72 patients) hypometabolism in the left temporal cortex compared to ALS-FTD patients [18]. No hypermetabolism was identified anywhere.	—	—	—

sALS	Cistaro et al.'s C9ORF72 ALS study also included a comparison with sALS patients. In C9ORF72 ALS, hypermetabolism was seen in the midbrain, bilateral occipital cortex, globus pallidus, left middle temporal cortex, and left inferior temporal cortex compared to sALS. Hypometabolism was seen in bilateral anterior and posterior cingulate cortices, insula, caudate, thalamus, left frontal cortex, and superior temporal cortex [18]. A recent study by De Vocht et al. demonstrated lower metabolism in the perirolandic region and increased metabolism in the brainstem [33].	Cistaro et al. reported that ALS-FTD had hypermetabolism in the bilateral occipital cortex, left precentral cortex, left postcentral cortex, and superior temporal gyrus while hypometabolism was in the right orbitofrontal, prefrontal, anterior cingulate, and insular cortices when compared to sALS [18].	—	—

SOD1 ALS	De Vocht et al. observed C9ORF72 hypometabolism in the thalamus, posterior cingulate gyrus, postcentral gyrus, and precentral gyrus compared to SOD1 ALS. Hypermetabolism in the brainstem, lentiform nucleus and cerebellum was observed compared to SODI ALS [33].	—	Canosa et al.'s SOD1 study revealed (in sALS compared to SOD1) hypometabolism in the right precentral gyrus, right medial frontal gyrus, right paracentral lobule, and bilateral postcentral gyrus. No hypermetabolism was identified [22]. De Vocht et al. revealed no difference between the groups [33].	—

**Table 2:** Additional metabolic comparisons across ALS phenotypes beyond Table 1.

Comparison	Result	Reference
Bulbar vs. spinal-onset ALS	Cistaro et al.'s study on bulbar and spinal onset found no significant differences in metabolism [29]. Pagani et al.'s study showed hypometabolism in bulbar patients in the left motor and premotor cortices as compared to spinal patients, but no significant hypermetabolism was found [23]. Sala et al.'s study did find bulbar hypometabolism in the left anterior cingulate, although it was not enough to distinguish between the two groups when performing discriminatory analysis [32]. Canosa et al.'s study found bulbar hypometabolism in the precentral gyrus but no hypermetabolism [37].	[23, 29, 32, 37]

C9ORF72 ALS vs. non-C9ORF72 ALS	Van Laere et al.'s study compared C9ORF72 ALS to all other non-C9ORF72 ALS subjects they had. They found reduced metabolism in C9ORF72 as compared to non-C9ORF72 ALS in the anterior and posterior cingulate, posterior thalamus, right lateral frontal cortex, and right temporoparietal junction. No hypermetabolism was found. However, it is important to note that the researchers had to turn down their preset threshold for significance (*p* < 0.05) to find such hypometabolic results and that their original threshold found no significant differences [25].	[25]

TARDBP ALS vs. non-TARDBP ALS	Canosa et al.'s study on TARDBP ALS compared TARDBP ALS to a control ALS group, where the control ALS group did not have FTD, had no mutation in major ALS genes, and had no family history of ALS. Compared to this ALS control group, the TARDBP ALS exhibited hypometabolism in the right precentral gyrus, right postcentral gyrus, superior temporal gyrus, middle temporal gyrus, and insula [20].	[20]

Comparison of ALS with varying levels of impairment and ALS-FTD	Canosa et al. did an additional study where ALS patients were grouped into multiple categories. These categories were ALS with normal cognition, ALS with impaired cognition, ALS with impaired behavior, ALS with impaired cognition and behavior, and ALS-FTD. Metabolism was compared across these groups. When comparing ALS-FTD to ALS with normal cognition, ALS-FTD hypometabolism was found in the right cingulate gyrus, bilateral middle frontal gyri, right precentral gyrus, bilateral superior frontal gyrus, and bilateral inferior frontal gyri while hypermetabolism was found in the cerebellum and brachia pontis (middle cerebellar peduncle). Cognitive-only impaired ALS patients showed hypometabolism (when the significance threshold was decreased) in the left superior, middle and inferior frontal gyri, superior temporal gyrus, and uncus, with no hypermetabolism as compared with cognitively normal ALS patients. Cognitive and behaviorally impaired ALS compared to cognitively normal ALS exhibited hypometabolism in the bilateral superior and middle frontal gyri, bilateral anterior cingulate gyri, right cingulate gyrus, right medial frontal gyrus, bilateral inferior frontal gyri, right precentral gyrus, and right insula, while there was no hypermetabolism [43].	[43]

High vs. low motor burden	A study by Sennfält et al. compared patients with a high motor symptom burden to those with a low motor symptom burden. The burden was assessed with a scale of impairment that was applied to several regions of the body. In the high burden group, hypometabolism was seen in the insula and left lingual gyrus [21].	[21]

Genetic vs. nongenetic ALS	Liu et al. compared ALS cases with genetic mutations in known ALS causing genes to nongenetic ones. In the genetic group, hypometabolism was observed in the postcentral gyrus, middle occipital gyrus, and right precuneus, but no hypermetabolism was seen [36].	[36]

**Table 3:** Summary of chemical structure of neuroinflammation targeting PET probes along with their application to ALS imaging.

PET radiotracer	Chemical name and structure	Application in ALS	References
[^11^C]PBR28	[^11^C]PBR28 (aryloxianilide) N-acetyl-N-(2–[^11^C]methoxybenzyl)-2-phenoxy-5-pyridinamine 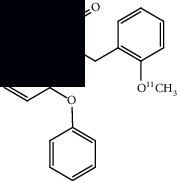	Increased uptake in motor regions and the precentral gyrus in ALS patients as compared with controls and the lung, spine, and brain (primarily the hindbrain) in mouse models.	[49–52]

[^11^C]PK11195	N-(sec-butyl)-1-(2-chlorophenyl)-N-(methyl-^11^C)isoquinoline-3-carboxamide 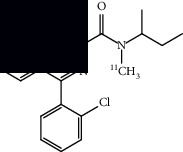	Several regions with increased uptake, including the motor cortex, frontal lobe, thalamus, occipital lobe, temporal lobe, pons, sensorimotor cortex, and cerebellum when compared to controls.	[55, 56]

[^18^F]DPA714	N,N-diethyl-2-(2-(4-(2-(fluoro-^18^F)ethoxy)phenyl)-5,7-dimethylpyrazolo[1,5-a]pyrimidin-3-yl)acetamide 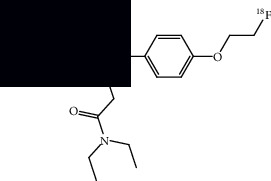	Increased primary motor cortex uptake in humans and increased brainstem uptake in mice models.	[52, 57]

[^11^C](l)-deprenyl-D2 or [^11^C]DED	(R)-N-(methyl-^11^C)-N-(1-phenylpropan-2-yl)prop-2-yn-1-amine-1,1-d2 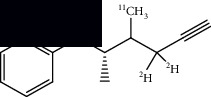	Increased uptake in pons and white matter, decreased uptake in parietal and temporal areas when compared to controls.	[59]

[^18^F]THK5351	(R)-1-(fluoro-^18^F)-3-((2-(6-(methylamino)pyridin-3-yl)quinolin-6-yl)oxy)propan-2-ol 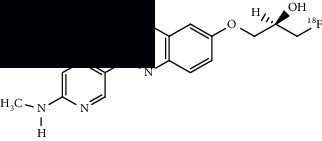	Multiple regions with increased uptake, such as the precentral gyri, motor cortex, and temporal lobes. Sample size is limited.	[60, 62]

[^11^C]KD2	N-((1s,3s)-adamantan-1-yl)-8-(methoxy-^11^C)-4-oxo-1-pentyl-1,4-dihydroquinoline-3-carboxamide 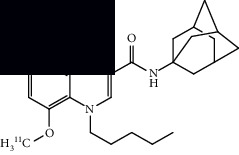	Demonstrated specific uptake in ALS postmortem spinal cord tissue.	[64]

[^18^F]RS-126	N-((1s,3s)-adamantan-1-yl)-1-(2-(2-(fluoro-^18^F)ethoxy)ethyl)-8-methoxy-4-oxo-1,4-dihydroquinoline-3-carboxamide 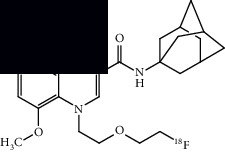	No specific binding to CB2 receptors in ALS postmortem spinal tissue.	[63]

[^11^C]RS-028	1-(2-Ethoxyethyl)-N-((1r,3r)-3-hydroxyadamantan-1-yl)-8-(methoxy-^11^C)-4-oxo-1,4-dihydroquinoline-3-carboxamide 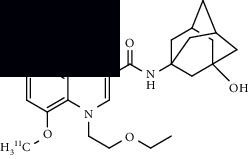	Increased and specific binding to ALS postmortem spinal tissue compared to control tissue.	[63]

**Table 4:** Summary of chemical structure of neuronal density imaging PET probes along areas of application for ALS research.

PET radiotracer	Chemical name and structure	Application in ALS	References
[^11^C]flumazenil	Ethyl 8-fluoro-5-(methyl-^11^C)-6-oxo-5,6-dihydro-4H-benzo[f]imidazo[1,5-a][1,4]diazepine-3-carboxylate 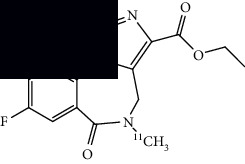	Decreased binding in mainly motor and frontal regions as compared to controls.	[6, 65, 66]

[^11^C]WAY100635	N-(2-(4-(2-(methoxy-^11^C)phenyl)piperazin-1-yl)ethyl)-N-(pyridin-2-yl)cyclohexanecarboxamide 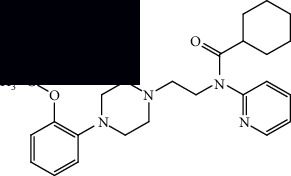	Decreased binding in several gyri and frontotemporal regions through SPM and VOI analysis as compared to controls.	[67]

[^18^F]FPEB	3-(Fluoro-^18^F)-5-(pyridin-2-ylethynyl)benzonitrile 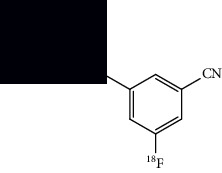	Increased binding in the hippocampus, striatum, and frontal cortex of mice models.	[49]

[^18^F]PSS232	(E)-3-(pyridin-2-ylethynyl)cyclohex-2-en-1-one O-(3-(2-(fluoro-^18^F)ethoxy)propyl) oxime 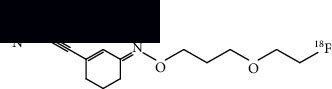	Increased binding in the striatum, hippocampus, and cortex in mice models and increased binding in the basal ganglia and frontal, motor, and temporal cortices of ALS postmortem tissue.	[48]

[^18^F]fallypride	N-((1-allylpyrrolidin-2-yl)methyl)-5-(3-(fluoro-^18^F)propyl)-2,3-dimethoxybenzamide 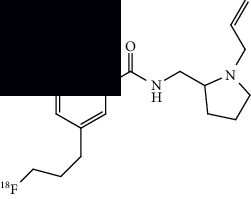	Reduced uptake in the superior frontal gyrus, nucleus accumbens, left temporal lobe, and angular gyrus in patients compared to controls.	[68]

[^18^F]SynVesT-1	(R)-4-(3-(fluoro-^18^F)phenyl)-1-((3-methylpyridin-4-yl)methyl)pyrrolidin-2-one 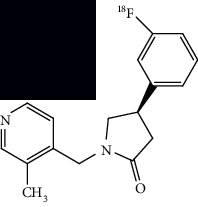	Decreased binding in the frontal lobe, anterior cingulate, hippocampus-insula region, and right temporal lobe in patients compared with controls.	[69]

[^18^F]OF-NB1	3-(4-(2-(Fluoro-^18^F)phenyl)butyl)-2,3,4,5-tetrahydro-1H-benzo[d]azepine-1,7-diol 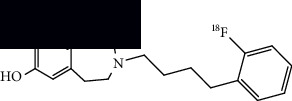	Decreased binding in postmortem frontal and motor cortex brain tissue compared to control tissue.	[70]

## Data Availability

There is no additional data associated with this manuscript other than what has been presented and cited in this review article.

## Additional Points

*Search Criteria*. Searches were done on PubMed. Prompts include “ALS PET,” “ALS SPECT,” “ALS FDG Bulbar,” “ALS C9ORF72 FDG,” and “ALS Neuroinflammation PET.”
